# Scientific literacy and preferred resources used by Latin American medical students during the COVID-19 pandemic: A multinational survey

**DOI:** 10.12688/f1000research.109398.2

**Published:** 2022-06-30

**Authors:** Bryan Nicolalde, Diego Añazco, Maria Jose Jaramillo-Cartwright, Ivonne Salinas, Aldo Pacheco-Carrillo, Saliha Hernández-Chávez, Gimena Moyano, Enrique Teran

**Affiliations:** 1 Colegio de Ciencias de la Salud, Universidad San Francisco de Quito, Quito, Ecuador; 2 Facultad de Medicina, Universidad Autonoma de Yucatán, Merida, Yucatan, Mexico; 3 Fundación H. Barceló, Instituto Universitario de Ciencias de la Salud, La Rioja, Argentina

**Keywords:** COVID-19; medical students; Latin America; critical scientific literature appraisal

## Abstract

**Background:** This study aims to identify the preferred sources for acquiring knowledge about COVID-19 and to evaluate basic knowledge on critical scientific literature appraisal in students from medical schools located in Spanish speaking countries in Latin America.

**Methods:** We designed an online survey of 15 closed-ended questions related to demographics, preferred resources for COVID-19 training, and items to assess critical appraisal skills. A snowball method was used for sampling. We conducted a descriptive analysis and Chi-squared tests to compare the proportion of correct identification of the concept of a preprint and a predatory journal when considering a) self-perceived level of knowledge, b) public vs private school, c) inclusion of a scientific literature appraisal subject in the curriculum, and d) progress in medical school.

**Results:** Our sample included 770 valid responses, out of which most of the participants included were from Mexico (n=283, 36.8%) and Ecuador (n=229, 29.7%). Participants preferred using evidence-based clinical resources (EBCRs) to learn more about COVID-19 (n=182, 23.6%). The preferred study design was case report/series (n=218, 28.1%). We found that only 265 participants correctly identified the concept of a preprint (34.4%), while 243 students (31.6%) correctly identified the characteristics of a predatory journal. We found no significant differences in the proportion of correct answers regardless of the self-perceived level of knowledge, progress in medical school, or scientific literature critical appraisal classes.

**Conclusion:** This study is novel in its approach of identifying sources of knowledge used by Latin American medical students and provides insights into the need to reinforce training in critical appraisal of scientific literature during medical school.

## Introduction

In December 2019, a cluster of cases of pneumonia of unknown origin was reported in Wuhan, China and since then, the virus has spread globally. On March 11, 2020, the World Health Organization (WHO) declared coronavirus disease 19 (COVID-19) as a pandemic. ^1^ Researchers throughout the globe have worked arduously to rapidly fill in the gaps of knowledge on this novel disease and this has resulted in an unprecedented surge in scientific production. ^2^ However, the pressure of produce science quickly can cause an overproduction of low-quality research. Notably, before the pandemic, it was estimated that approximately 85% of research was wasted due to flaws in design, methodology or interpretation of the data, and these challenges have been augmented due to the time constraints and poor research infrastructure during the COVID-19 pandemic. ^3^^,^^4^

The role of medical students has been diverse throughout history, from being participatory agents in the evolution of medical curricula to being agents of promotion, prevention and treatment of diseases. ^5^^,^^6^ Medical students have helped during previous disasters and emergencies such as the Spanish flu outbreak of 1918 and the 1952 polio epidemic in Denmark; and they might have an important role during the COVID-19 pandemic through essential tasks such as assistance in clinical care, telemedicine, and fighting against misinformation. ^7^^,^^8^

To provide high-quality healthcare services, future physicians should develop abilities to critically assess research to inform evidence-based decisions during their career, including critical appraisal of scientific literature within the curriculum of medical schools and promoting involvement in research opportunities could help students develop these skills. ^9^^–^^12^ Notably, the percentage of student authorship in published articles has increased importantly throughout the years. ^13^

Due to their role, and the unprecedent low quality scientific information, it is indispensable that medical students have enough skills to analyse scientific data. In this study, we intend to identify preferred resources used by medical students in Spanish speaking countries in Latin America to obtain information about COVID-19 and evaluate basic critical appraisal skills through a self-report online survey.

## Methods

### Ethical statement

This study was approved by the Independent Review Board at Universidad San Francisco de Quito (2020-033M). Although data was anonymous and did not include identifying information, electronic informed consent was requested before the survey was collected. Participation was voluntary, and medical students were informed that data from the survey would be used for analysis and scientific publication. Participants did not receive any compensation from participating in the study.

### Study design

This was a multinational cross-sectional study based on a self-report online survey. Participants were medical students currently enrolled in a medical school in a Spanish-speaking country in Latin America. The inclusion criteria required for participation were that they were aged 18 or older, with active student status in a medical school based in a Spanish-speaking country in Latin America, and with Spanish language fluency. Exclusion criteria were non-active student status, medical students from a country where Spanish is not the primary language, and previous participation in the survey.

As we intended to include participants from multiple countries and achieve significant variation in characteristics within our purposive sample, a link to the survey and an accompanying standardized text explaining the purpose of the survey was sent via e-mail from the authors to the initial “point of contact” in each country for further distribution following a snowballing approach. The link to the survey was then shared through different social networks by authors to medical students from their own and other medical schools, and authors instructed participants to share the link with their peers. The survey was distributed to students from different academic years to gather a diverse sample in terms of progress in medical school. We also actively tried to contact students from as many countries in Latin America as possible through e-mails sent to the medical students’ organizations publicly available to ask them to distribute the survey to their members. Data collection took place from October 22 to November 6, 2020. During the first week after the survey was released we obtained a huge amount of the answers, but then there was a very low number of new responses daily, therefore we decided to shorten the period for data gathering.

### Survey design

We designed an electronic survey using the Google Forms web application. The survey consisted of fifteen closed-ended questions to gather basic demographic information and evaluate resources used to obtain information on COVID-19 and explore knowledge and attitudes that medical students had in terms of scientific information appraisal. The survey was developed by employing a conceptual framework, in which previous knowledge and experiences that authors had with publications related to medical education, as well as their personal experiences as medical students or faculty, guided the creation of questions. ^6^^,^^14^ Authors were invited to submit potential questions to a shared online document, which then underwent revision through a telematic session by all authors to determine the questions that would be included in the survey. The panel of authors included active students, alumni, and faculty with vast experience in medical education and research.

The survey was developed in Spanish and was reviewed by all authors to ensure that it was appropriate despite regional language variations. In addition, two independent academic advisors reviewed the survey for clarity. At this stage, no changes were required. Then, the survey was piloted in medical students from Ecuador (n=5), Mexico (n=3), and Argentina (n=2), who were mainly friends of the authors, to get their input and identify confusing or unclear questions. These surveys were not included in the analysis. Pretest resulted in minor changes, mostly related to grammar and punctuation After incorporating the feedback, we deployed the survey. As the survey was originally designed and applied in Spanish, we have included a translation to English which was performed by an author (BN) with certified proficiency and then by a native speaker who did not participate in the study (The original version in Spanish and a translated version to English are available as extended data). ^14^

### Statistical analysis

Data captured through the Google Forms web application was exported directly to Microsoft Excel (v. 16.58). An initial descriptive analysis of the responses to the survey to determine demographic characteristics and preferred resources used to gather COVID-19 related information was performed. All variables obtained in the results of the survey were analyzed. There was no missing data as the setting in the online survey did not allow for the submission of incomplete forms. We analyzed the difference in the proportion of correct identification of the concept of a preprint and predatory journals when comparing students based on their self-reported level of knowledge, progress in medical school, public vs. private status of medical school, and inclusion of a scientific literature critical appraisal subject during their curriculum. For data analysis, we decided to group low-medium and advanced-experts’ responses in the self-reported level of knowledge and first-second quarter and third-fourth quarter in progress in medical school responses to create dichotomized variables. Due to the categorical nature of our data, we decided to use Chi-squared tests. A one-tailed alpha value of 0.05 was set. All our statistical analyses were conducted on IBM SPSS version 25 (IBM. Corp., Armonk, N.Y., USA).

### Trustworthiness

By incorporating different stakeholders in medical education (active junior and senior students, alumni, and faculty), and researchers from different countries, we could design a study that contained different valuable perspectives on the critical appraisal of scientific information.

Although ET is a professor in one of the medical schools where the survey was conducted, participants were not aware of his role in the research. No economic or academic incentive was offered by any of the authors to the participants.

After data gathering, BN and DA performed an initial statistical analysis and shared the results with the other authors. A researcher not involved in this study was invited to validate the statistical analysis. Additionally, all authors had access to the database and agreed with the final data analysis and processing.

## Results

There were 770 valid responses to the survey (Underlying data: Survey results-Scientific literacy and preferred resources used by Latin American medical students during COVID-19 pandemic). ^14^ Almost two-thirds of the participants were females (n=487, 63.2%). Most of the participants were from Mexico (n=283, 36.8%) and Ecuador (n=229, 29.7%). Students equally attended private (n=388, 50.4%) or public universities (n=382, 49.6%). We also found a similar distribution in terms of completed progress in medical school: 24.0% (n=185) in the first quarter, 24.3% (n=187) in the second quarter, 24.9% (n=192) in the third quarter, and the remaining 26.8% (n=206) in the final quarter of medical school. Distribution by gender and country is shown in Table 1.

**Table 1.** Distribution by gender of medical students from Latin America.

	Female (n=487)	Male (n=278)
Argentina	15 (3,08)	3 (1,08)
Bolivia	9 (1,85)	1 (0,36)
Chile	18 (3,70)	23 (8,27)
Colombia	8 (1,64)	8 (2,88)
Costa Rica	1 (0,21)	0 (0,00)
Dominican Republic	4 (0,82)	0 (0,00)
Ecuador	156 (32,03)	73 (26,26)
El Salvador	5 (1,03)	1 (0,36)
Guatemala	52 (10,68)	14 (5,04)
Honduras	21 (4,31)	26 (9,35)
Mexico	168 (34,50)	114 (41,01)
Nicaragua	1 (0,21)	0 (0,00)
Panama	8 (1,64)	4 (1,44)
Paraguay	8 (1,64)	0 (0,00)
Peru	8 (1,64)	4 (1,44)
Uruguay	1 (0,21)	2 (0,72)
Venezuela	4 (0,82)	5 (1,80)

Data are presented as the number (%). 5 participants answered “other”: one from Chile, one from Guatemala, two from Honduras, and one from Mexico.

Most medical students considered that they had a low or medium level of knowledge about COVID-19 (n=539, 70.0%), while 231 participants reported an advanced or expert level (30.0%).

Overall, medical students preferred using evidence-based clinical resources (EBCRs), such as UpToDate or Medscape, to learn more about COVID-19 (n=182, 23.6%), followed by academic journals (n=171, 22.2%), and open access courses provided by entities such as the World Health Organisation (WHO) (n=141, 18.3%). Our survey responses were grouped according to the different sub-regions of Latin America ( Table 2).

**Table 2.** Sources for COVID-19 training amongst medical students in Latin America.

	North America (n=283)	Central America and the Caribbean (n=140)	South America (n=347)
Academic journals	71 (25,09)	35 (25,00)	65 (18,73)
Evidence-based clinical resources	39 (13,78)	15 (10,71)	128 (36,89)
Continuous medical education programs of international universities	35 (12,37)	37 (26,43)	44 (12,68)
Continuous medical education programs of local universities	45 (15,90)	21 (15,00)	60 (17,29)
WHO, PAHO medical education programs	76 (26,86)	27 (19,29)	38 (10,95)
Peer discussion with classmates	10 (3,53)	2 (1,43)	9 (2,59)
Others	7 (2,47)	3 (2,14)	3 (0,86)

The number and (percentage) of medical students who chose each source for COVID-19 training is shown.

**Figure 1.**
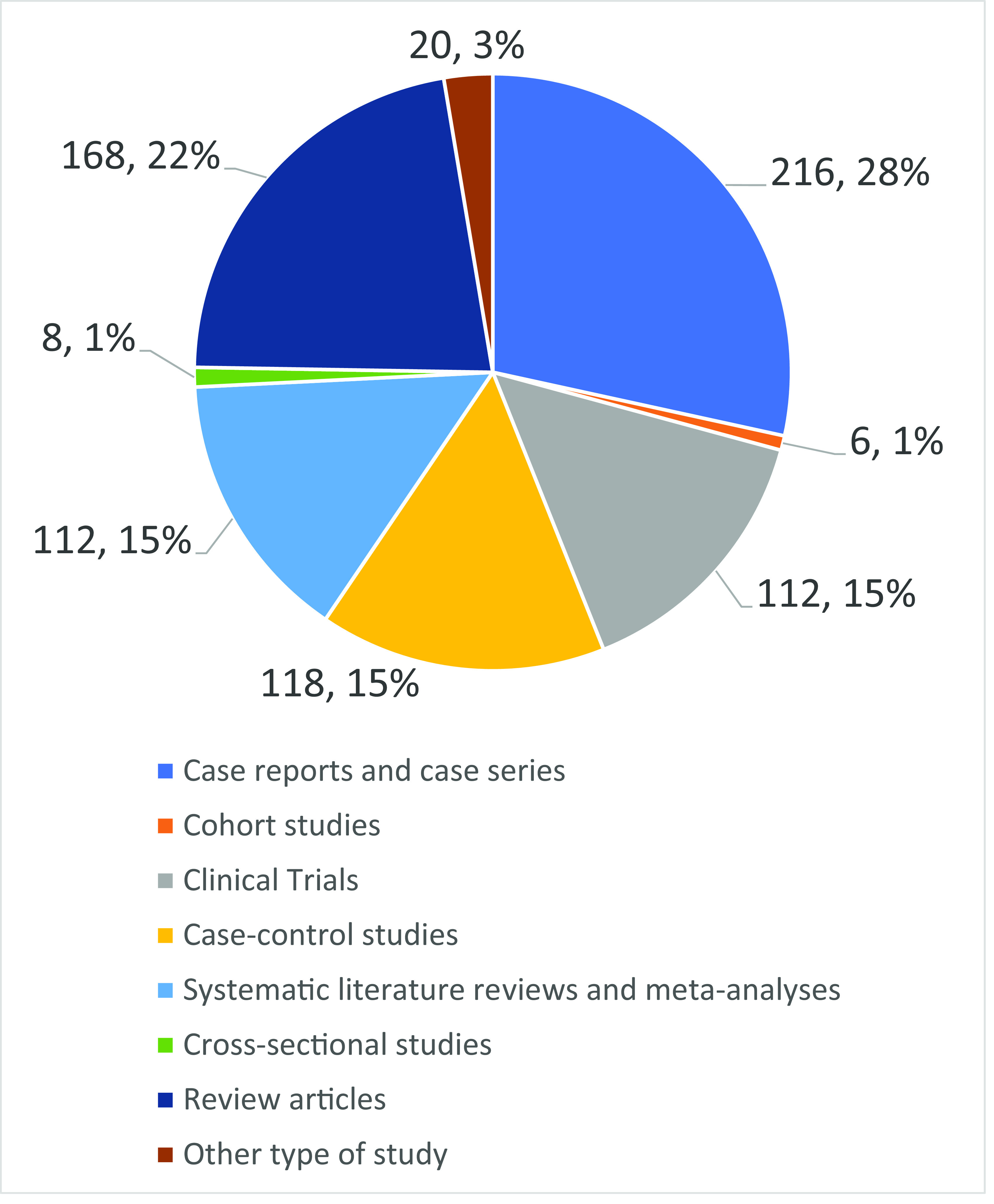
Preferences of type of article among medical students of Americas.

Regarding the type of article, medical students preferred using case reports or case series (n=218, 28.1%) to learn more about COVID-19, followed by review articles (n=168, 21.8%) ( Figure 1).

The critical factor that medical students considered when reading a scientific article was most frequently the affiliation of the authors (n=366, 47.5%), followed by the journal of publication (n=176, 22.85%), the type of study (n=138, 17.92%), and the publication status (preprint vs. published) (n=44, 5.71%).

In terms of receiving news related to the COVID-19 pandemic, medical students preferred official websites of organizations such as the WHO (n=221, 28.7%), followed by scientific journals (n=185, 24.0%), and medical platforms (n=128, 16.6%) ( Table 3).

**Table 3.** Comparison between medical students of different regions of America and main source of COVID-19 news.

	North America (n=283)	Central America and Caribbean (n=140)	South America (n=347)
Social media	42 (14,84)	22 (15,71)	60 (17,29)
WHO, PAHO, EPI-WIN, among others	107 (37,81)	43 (30,71)	71 (20,46)
Scientific community and news anchors opinions on television	35 (12,37)	18 (12,86)	33 (9,51)
NGOs summaries and briefs	4 (1,41)	7 (5,00)	10 (2,88)
Scientific journals	79 (27,92)	30 (21,43)	76 (21,90)
Medical platforms	14 (4,95)	19 (13,57)	95 (27,38)
Others	2 (0,71)	1 (0,71)	2 (0,58)

The number and (percentages) of medical students that chose each source as the one they use for COVID-19 news is shown.

Additionally, relationship between percentage of undergraduate studies completed, self-perceived knowledge on COVID-19 and, source of preference for COVID-19 information were performed ( Table 4).

**Table 4.** Relationship between percentage of undergraduate studies completed, self-perceived knowledge on COVID-19 and, source of preference for COVID-19 information.

		Percentage of completed studies				Self-perceived knowledge in COVID-19	
		Less than 25% (n=185)	Between 25% and less than 50% (n=187)	Between 50% and less than 75 (n=192)	Over 75% (n=206)	Low-medium (n=539)	Advanced-expert (n=231)
About your COVID-19 training and skill building, what kind of sources do you use primarily?	Academic journals	22 (11,89)	49 (26,20)	48 (25,00)	52 (25,24)	119 (22,08)	52 (22,51)
	Medical platforms	30 (16,22)	32 (17,11)	52 (27,08)	68 (33,01)	128 (23,75)	54 (23,38)
	Continuous medical education programs of international universities	32 (17,30)	30 (16,04)	23 (11,98)	31 (15,05)	78 (14,47)	38 (16,45)
	Continuous medical education programs of local universities	44 (23,78)	37 (19,79)	26 (13,54)	19 (9,22)	96 (17,81)	30 (12,99)
	WHO, PAHO medical education programs	46 (24,86)	30 (16,04)	36 (18,75)	29 (14,08)	95 (17,63)	46 (19,91)
	Peer discussion with classmates	11 (5,95)	3 (1,60)	3 (1,56)	4 (1,94)	18 (3,34)	3 (1,30)
	Others	0 (0,00)	6 (3,21)	4 (2,08)	3 (1,46)	5 (0,93)	8 (3,46)
What is the main platform you use to get updates about COVID-19	Social media	35 (18,92)	38 (20,32)	27 (14,06)	24 (11,65)	96 (17,81)	28 (12,12)
	WHO, PAHO, EPI-WIN, among others	66 (35,68)	55 (29,41)	56 (29,17)	44 (21,36)	144 (26,72)	77 (33,33)
	Scientific community and news anchors opinions on television.	31 (16,76)	22 (11,76)	21 (10,94)	12 (5,83)	71 (13,17)	15 (6,49)
	NGOs summaries and briefs	9 (4,86)	7 (3,74)	3 (1,56)	2 (0,97)	20 (3,71)	1 (0,43)
	Scientific journals and papers platforms	29 (15,68)	50 (26,74)	42 (21,88)	64 (31,07)	113 (20,96)	72 (31,17)
	Medical platforms	13 (7,03)	13 (6,95)	42 (21,88)	60 (29,13)	93 (17,25)	35 (15,15)
	Others	2 (1,08)	2 (1,07)	1 (0,52)	0 (0,00)	2 (0,37)	3 (1,30)
What kind of academic papers have you used the most in order to have COVID-19 updated information?	Case/case series report	53 (28,65)	52 (27,81)	54 (28,13)	57 (27,67)	146 (27,09)	70 (30,30)
	Clinical trials	34 (18,38)	34 (18,18)	27 (14,06)	17 (8,25)	77 (14,29)	35 (15,15)
	Case control studies	40 (21,62)	29 (15,51)	22 (11,46)	27 (13,11)	77 (14,29)	41 (17,75)
	Systematic literature revies/Methanalysis	19 (10,27)	25 (13,37)	33 (17,19)	45 (21,84)	82 (15,21)	40 (17,32)
	Review articles	26 (14,05)	38 (20,32)	49 (25,52)	55 (26,70)	131 (24,30)	37 (16,02)
	Cohort studies	2 (1,08)	2 (1,07)	2 (1,04)	0 (0,00)	5 (0,93)	1 (0,43)
	Cross-sectional studies	2 (1,08)	2 (1,07)	2 (1,04)	2 (0,97)	4 (0,74)	4 (1,73)
	Other type of articles	9 (4,86)	5 (2,67)	3 (1,56)	3 (1,46)	17 (3,15)	3 (1,30)
What is the main criteria you take into account when reading an article or paper	Study design	14 (7,57)	34 (18,18)	33 (17,19)	57 (27,67)	90 (16,70)	48 (20,78)
	Author affiliation	4 (2,16)	1 (0,53)	2 (1,04)	3 (1,46)	5 (0,93)	5 (2,16)
	Endorsement from institutions	127 (68,65)	84 (44,92)	83 (43,23)	72 (34,95)	250 (46,38)	116 (50,22)
	Publishing status (peer review vs preprint)	5 (2,70)	9 (4,81)	13 (6,77)	17 (8,25)	31 (5,75)	13 (5,63)
	Number of references	5 (2,70)	12 (6,42)	9 (4,69)	4 (1,94)	20 (3,71)	10 (4,33)
	Journal where it is published	27 (14,59)	44 (23,53)	52 (27,08)	53 (25,73)	138 (25,60)	38 (16,45)
	Others	3 (1,62)	3 (1,60)	0 (0,00)	0 (0,00)	5 (0,93)	1 (0,43)

We found that approximately a third of the participants correctly identified the concept of a preprint (n=265, 34.4%), while 243 students (31.6%) correctly identified the characteristic of a predatory journal. On the other hand, 553 students (71.8%) correctly identified the concept of an open-access journal.

Two-thirds of the participants (n=526, 68.3%) had a subject focused on scientific literature critical appraisal as a part of their curriculum during medical school.

We conducted chi-squared tests to identify factors that were associated with a significant difference in the proportion of students that correctly identified the concept of a preprint or predatory journals (Extended data: Frequencies of answers and results of Chi-squares performed). ^14^ We did not find a significant difference when comparing participants in terms of self-reported level of knowledge (low/medium vs advanced/expert) in the chance of correctly identifying the concept of a preprint (df=1, χ ^2^=0.632, P=0.456) or a predatory journal (df=1, χ ^2^=0.435, P=0.554).

Participants from public schools correctly identified the concept of predatory journals (n=142, 37.1%) more frequently than students that attended a private medical school (n=101, 26.0%) (df=1, χ ^2^=11.063, P<0.01); however, there was not a significant difference while identifying the concept of a preprint (df=1, χ ^2^=2.805, P=0.094).

Additionally, we did not find significant differences in the correct identification of preprint (df=1, χ ^2^=0.502, P=0.514) or predatory journal (df=1, χ ^2^=0.694, P=0.406) concepts regardless of the inclusion of a scientific literature critical appraisal subject during medical school. Finally, the proportion of participants that answered correctly was not significantly different for preprints (df=3, χ ^2^=7.124, P=0.068) and predatory journals (df=3, χ ^2^=5.741, P=0.125) despite the progress in medical school (first-second quarter vs third-four quarter).

## Discussion

Despite the presence of data from some countries showing an increasing proportion of female medical students, no regional report of the general characteristics of medical students in Latin America has been performed recently. Other cross-sectional studies in Latina American medical schools showed a similar distribution in terms of gender. ^15^^,^^16^ Overall, most medical students perceived that their knowledge about COVID-19 was either low or medium independently of the current academic year they belonged. This differs from what could be expected in previous studies in which students from superior years were more knowledgeable on COVID-19. ^17^ Medical students who were more advanced in their career (>50%) preferred evidence-based clinical resources (EBCR) for train and skill building. Notable, regardless of self-perceived knowledge of COVID-19, medical students preferred EBCR ( Table 4). This result is consistent with previous research that shows the popularity of these resources within the medical community. ^15^^,^^16^ The use of evidence-based electronic resources has been associated with reduced lengths of stay and mortality rates, and it has resulted in improved performances in standardized examinations. ^18^^,^^19^ Additionally, these results represent that with the progress of their career medical students improve their abilities to choose the main source for skill building.

Most of participants preferred getting news related to the pandemic on official websites by entities such as the WHO and the Centre for Disease Control (CDC), which could be a way to combat the current misinformation outbreak (infodemic). Social media platforms provide users with immediate access to massive amounts of content and are the perfect medium to spread questionable information easily. ^20^ A study showed that fake news is easily spread through Latin American countries through social media platforms, and fact-checking could be a potential solution to fight back against the infodemic. ^21^

Notably, participants mentioned a preference for case series/reports to learn more about COVID-19, followed by narrative reviews and case-control studies ( Table 4). Only 112 participants (15%) preferred systematic literature reviews (SLRs) or metanalyses. The importance of addressing this trend relies on the level of evidence that well-conducted SLRs and metanalysis have compared to case series/reports, which has traditionally been portrayed on the top and the bottom of the evidence pyramid, respectively. ^22^^,^^23^ It would be expected that medical students who had exposure to a subject focused on critical appraisal skills during their formation would choose study designs higher among the evidence pyramid, however, our results did not show a significant difference. These could be partially attributed to the fact that during the start of the pandemic most of the articles published were retrospective studies or case reports focusing on patients’ characteristics. ^2^ Remarkably, we found that regardless of the progress in medical school, self-perceived level of knowledge about COVID-19, and the inclusion of a critical appraisal subject in the curriculum, most of the participants could not correctly identify the concept of a preprint or a predatory journal, despite the enormous surge in preprint production during this pandemic. ^24^ As well, in our study, only 44 participants (5.71%) chose the publication status (preprint vs published article) as a critical factor when reading an article. Overall, regardless percentage of career completed, or self-perceived level of COVID-19 knowledge medical students take into consideration as main critical factor to read an article endorsement from institutions ( Table 4). This is concerning as medical students might fail to recognize that preprints are preliminary reports that have not been peer-reviewed and might contain faulty or low-quality information. ^25^

Additionally, as medical students are more frequently involved in research, they could be potential targets for predatory journals that promise easy and rapid pathways to publish, through unethical practices, which could further contribute to the infodemic. ^13^ As expected, predatory journals have exploited the uncertainty and prolonged reviewing and editing processes by multiple scholarly journals due to the pandemic to increase their profits. ^26^^–^^28^ A previous study conducted in the Kingdom of Saudi Arabia and New Zealand also revealed a poor understanding of medical students regarding predatory journals. ^29^

The main limitation to our study is the imbalanced sample, which resulted in multiple countries having few participants. This could be related to the short time that the survey was available or ineffective sampling strategies for some specific countries. The chain-referral sampling method employed, despite its pragmatism, is a significant limitation, as the sampling is non-random due to the referral process, in which the sample is dependent on the researchers’ contacts, which is reflected by the significantly higher proportion of participants from Mexico and Ecuador. As the survey was available online, of course there was the possibility of participants outside the target population to complete it, however, this bias was dismissed by employing a chain referral model among medical students. As well, there is uncertainty of whether the sample is representative of the target population. Furthermore, participants could be susceptible to subject bias and might have elected responses that seemed more appropriate for a medical student.

This study is novel in its approach to identify sources used by Latin American medical students to learn about COVID-19 and to evaluate critical appraisal skills used to interpret new scientific information. Additionally, even though we had difficulties in sampling medical students from certain nationalities, we did recruit an equative number of medical students representing different stages of medical degree completion. Our results support the need to reinforce training in critical appraisal of scientific literature during medical school. ^30^^,^^31^

## Conclusions

Throughout history medical students have an important role, and the COVID-19 crisis is not an exception. Our study found that most of medical students have good behaviors regarding acquire new information related to COVID-19 such as using evidence-based clinical resources to learn about COVID-19 and official websites of recognised organizations to receive news. However, they have problems in terms of correctly identifying predatory journals or preprint articles independently of the region, self-perceived level of knowledge, progress in medical school, or literature critical appraisal classes. Besides, these students prefer a study design that is not at the top of the evidence pyramid. Our findings could provide insights into the need to reinforce training in critical appraisal of scientific literature during medical school in Latin America.

## Data availability

### Underlying data

Open Science Framework (OSF). Scientific literacy and preferred resources used by Latin American medical students during COVID-19 pandemic. https://doi.org/10.17605/OSF.IO/7MS64. ^14^

This project contains the following underlying data:

• Survey results-Scientific literacy and preferred resources used by Latin American medical students during COVID-19 pandemic. (These data contain the answers obtained from the surveys that were applied in medical students about scientific literacy and preferred resources).
• Frequencies of answers and results of Chi-squares performed. (These data include how data was analysed)

Data are available under the terms of the Creative Commons Zero “No rights reserved” data waiver (CC0 1.0 Public domain dedication).

### Extended data

Open Science Framework (OSF). Scientific literacy and preferred resources used by Latin American medical students during COVID-19 pandemic. https://doi.org/10.17605/OSF.IO/7MS64. ^14^

This project contains the following extended data:

• Infodemic survey English version. (These data include the English version of the survey that was applied to medical students from Latin American Countries)
• Infodemic survey Spanish version. (These data include the original Spanish version of the study that was used in medical students from Latin American Countries)

Data are available under the terms of the Creative Commons Zero “No rights reserved” data waiver (CC0 1.0 Public domain dedication).
