# Supplementary material for: Scientific literacy and preferred resources used by Latin American medical students during the COVID-19 pandemic: A multinational survey
Source: F1000Res. 2022 Jun 30;11:341. Originally published 2022 Mar 21. [Version 2] doi: 10.12688/f1000research.109398.2 (PMC9296994; doi:10.12688/f1000research.109398.2)
Supplement: Peer review history

# Peer review: Reviewer response for version 2

**DOI:** 10.5256/f1000research.135452.r142785

**Published:** 2022-07-19

**Authors:** Chung-Ying Lin

**Affiliations:** 1 Institute of Allied Health Sciences, College of Medicine, National Cheng Kung University, Tainan, Taiwan

I feel that the revision is satisfactory and I have no more comments.

Is the work clearly and accurately presented and does it cite the current literature?

Yes

If applicable, is the statistical analysis and its interpretation appropriate?

Partly

Are all the source data underlying the results available to ensure full reproducibility?

Yes

Is the study design appropriate and is the work technically sound?

Yes

Are the conclusions drawn adequately supported by the results?

Partly

Are sufficient details of methods and analysis provided to allow replication by others?

Partly

Reviewer Expertise:

Psychosocial

I confirm that I have read this submission and believe that I have an appropriate level of expertise to confirm that it is of an acceptable scientific standard.

# Peer review: Reviewer response for version 2

**DOI:** 10.5256/f1000research.135452.r142786

**Published:** 2022-07-04

**Authors:** Fernando Spilki

**Affiliations:** 1 One Health Laboratory, Feevale University, Novo Hamburgo, Brazil

The authors made changes that resolved minor drawbacks found in the first version of the article. In my opinion, the current version is adequate, the information provided is relevant and the reading of the text leads to important insights on aspects of medical education and the use of adequate information sources by medical students in Latin America.

Is the work clearly and accurately presented and does it cite the current literature?

Yes

If applicable, is the statistical analysis and its interpretation appropriate?

Yes

Are all the source data underlying the results available to ensure full reproducibility?

Yes

Is the study design appropriate and is the work technically sound?

Yes

Are the conclusions drawn adequately supported by the results?

Yes

Are sufficient details of methods and analysis provided to allow replication by others?

Yes

Reviewer Expertise:

Virology, infectious diseases

I confirm that I have read this submission and believe that I have an appropriate level of expertise to confirm that it is of an acceptable scientific standard.

# Peer review: Reviewer response for version 1

**DOI:** 10.5256/f1000research.120893.r137749

**Published:** 2022-05-18

**Authors:** Fernando Spilki

**Affiliations:** 1 One Health Laboratory, Feevale University, Novo Hamburgo, Brazil

The issue of information quality proved to be extremely relevant in the context of the COVID-19 pandemic, especially during the first year. An infinity of obscure sources of information, including from social networks to a profusion of material published without any type of quality review, gave the opportunity for untruths to be raised to the level of scientific knowledge, greatly harming the fight against the pandemic in many scenarios.

 In this sense, the study becomes relevant and very interesting to assess the levels of understanding of medical students at different levels of training, about what they consider reliable sources of information, and how they judge dubious sources.

 From the point of view of minor points that require further investigation, it would be interesting to clarify and discriminate more clearly how students have judged the diverse types of information sources, depending on their level of completion of the medical curriculum, from the beginning to the end of graduation, and how this does evolves or not throughout.

Is the work clearly and accurately presented and does it cite the current literature?

Yes

If applicable, is the statistical analysis and its interpretation appropriate?

Yes

Are all the source data underlying the results available to ensure full reproducibility?

Yes

Is the study design appropriate and is the work technically sound?

Yes

Are the conclusions drawn adequately supported by the results?

Yes

Are sufficient details of methods and analysis provided to allow replication by others?

Yes

Reviewer Expertise:

Virology, infectious diseases

I confirm that I have read this submission and believe that I have an appropriate level of expertise to confirm that it is of an acceptable scientific standard, however I have significant reservations, as outlined above.

# Response

**Published:** 2022-06-23

**Authors:** Enrique Teran

**Affiliations:** Universidad San Francisco de Quito, Ecuador

- From the point of view of minor points that require further investigation, it would be interesting to clarify and discriminate more clearly how students have judged the diverse types of information sources, depending on their level of completion of the medical curriculum, from the beginning to the end of graduation, and how this does evolves or not throughout.

 We thank you Professor Spilki for his comments and regarding his suggestion, in the new version, this information has been added to new table 4.

# Peer review: Reviewer response for version 1

**DOI:** 10.5256/f1000research.120893.r137265

**Published:** 2022-05-12

**Authors:** Chung-Ying Lin

**Affiliations:** 1 Institute of Allied Health Sciences, College of Medicine, National Cheng Kung University, Tainan, Taiwan

The manuscript can be improved with the following considerations:

1. Please elaborate on how the 15 closed-ended questions were generated. It is unclear where the 15 questions come from. Did the authors conduct a literature review to identify potential items and then reduce the items via expert panels? It is also unclear what the following procedure is: " *The survey was designed specifically for this study by the authors; they included active students, alumni, and faculty with vast experience in medical education and research.*" Is this an expert panel that discusses the survey questions? Or, is this an informal chat between the authors and all the other stakeholders? Accordingly, it is unclear what the purpose of pilot testing on the medical students is. The authors mentioned that they wanted to get "input" from the piloted medical students. However, it is unclear what "input" the authors wanted to get. I suspect that the authors did not provide proper instructions to these students and thus they only obtained some minor comments. Also, it is unclear what the background of these students was (e.g., their gender, their studying progress).
2. The authors only used chi-squared tests for all the inferential statistics. Only using chi-squared tests has the problem of not controlling potential confounders. Given that the sample size was large, the authors should be able to apply multivariable statistics that control potential confounders (e.g., multivariable logistic regression).
3. I wonder how representative the sample was. Specifically, does the demographic distribution of the present sample similar to these surveyed countries? I found that it is interesting to have a higher proportion of females. 
4. Following the previous comment, I wonder how the authors ensure if the participants were the target participants. For example, maybe a student who was not a medical student received the email and completed the survey. Can the authors catch these invalid responses?
5. Given that health literacy on COVID-19 is the key information in the present study, I think that the authors should examine other potential factors on the COVID-19 literacy. Specifically, why don't the authors examine if the resources obtaining COVID-19 knowledge is a factor explaining the student's COVID-19 knowledge? Similarly, it would be interesting to examine if different types of articles the students read play a role in their COVID-19 knowledge. 
6. I disagree with the statement "The main limitation to our study is the sample size". Specifically, the entire sample size is good, and the problem is that the authors had an imbalanced sample size. After all, the authors did not separate the participants into different countries for the data analysis. Therefore, the authors did not have the problem of a small sample size in statistics. 

Is the work clearly and accurately presented and does it cite the current literature?

Yes

If applicable, is the statistical analysis and its interpretation appropriate?

Partly

Are all the source data underlying the results available to ensure full reproducibility?

Yes

Is the study design appropriate and is the work technically sound?

Yes

Are the conclusions drawn adequately supported by the results?

Partly

Are sufficient details of methods and analysis provided to allow replication by others?

Partly

Reviewer Expertise:

psychosocial

I confirm that I have read this submission and believe that I have an appropriate level of expertise to confirm that it is of an acceptable scientific standard, however I have significant reservations, as outlined above.

# Response

**Published:** 2022-06-23

**Authors:** Enrique Teran

**Affiliations:** Universidad San Francisco de Quito, Ecuador

- Please elaborate on how the 15 closed-ended questions were generated. It is unclear where the 15 questions come from. Did the authors conduct a literature review to identify potential items and then reduce the items via expert panels? It is also unclear what the following procedure is: "The survey was designed specifically for this study by the authors; they included active students, alumni, and faculty with vast experience in medical education and research." Is this an expert panel that discusses the survey questions? Or, is this an informal chat between the authors and all the other stakeholders? Accordingly, it is unclear what the purpose of pilot testing on the medical students is. The authors mentioned that they wanted to get "input" from the piloted medical students. However, it is unclear what "input" the authors wanted to get. I suspect that the authors did not provide proper instructions to these students and thus they only obtained some minor comments. Also, it is unclear what the background of these students was (e.g., their gender, their studying progress).

Thank you for your comment. This has been improved in v2 of our manuscript. Briefly, potential item were obtained either from literature or previous experience. As most of the authors were not experts in the field, the survey questions were defined through informal chat between the authors and other stakeholders. The pilot testing was a strategy to ensure the questions were fully understood or to get feedback to improve it. In fact, they were very well instructed about their role, not only in the answer as potential participants.

  

- The authors only used chi-squared tests for all the inferential statistics. Only using chi-squared tests has the problem of not controlling potential confounders. Given that the sample size was large, the authors should be able to apply multivariable statistics that control potential confounders (e.g., multivariable logistic regression).

We thank you Professor Chung-Ying Li for his suggestion. However, as all our data is qualitative, after consulting with our advisor in statistics, could not find any added value to applying multivariable analysis.

  

- I wonder how representative the sample was. Specifically, does the demographic distribution of the present sample similar to these surveyed countries? I found that it is interesting to have a higher proportion of females. 

As was described in the *Results *section, according to our knowledge, the distribution of the data is consistent with other Cross-Sectional studies performed in Latin America Region. Therefore, yes, our sample was representative. In the same sense, we initially had the same concern that reviewer 1 regarding the gender distribution. However, once again, in most of these kinds of studies, females are more active in response.

  

- Following the previous comment, I wonder how the authors ensure if the participants were the target participants. For example, maybe a student who was not a medical student received the email and completed the survey. Can the authors catch these invalid responses?

 

 It is important to mention that although the collection of data was performed through an online survey, and the risk of someone outside the target population might answer the survey, we consider that this risk was reduced by employing a chain-referral method with other medical students.

- Given that health literacy on COVID-19 is the key information in the present study, I think that the authors should examine other potential factors on COVID-19 literacy. Specifically, why don't the authors examine if the resources obtaining COVID-19 knowledge is a factor explaining the student's COVID-19 knowledge? Similarly, it would be interesting to examine if different types of articles the students read play a role in their COVID-19 knowledge. 

To explore other factors that could contribute to Covid-19 literary among medical students, we perform an additional descriptive analysis where we make a relationship between the percentage of undergraduate studies completed, self-perceived knowledge on COVID-19, and source of preference for COVID-19 information that is summarized in *Table 4 *and discussion section. This analysis was performed to study if the behavior among medical students changes along with the progress of their career or the level of auto perception in knowledge choosing better sources to acquire medical knowledge.

  

- I disagree with the statement "The main limitation to our study is the sample size". Specifically, the entire sample size is good, and the problem is that the authors had an imbalanced sample size. After all, the authors did not separate the participants into different countries for the data analysis. Therefore, the authors did not have the problem of a small sample size in statistics.

We agree with Professor Chung-Ying Li that the correct term is not a sample size, instead, we change the term to *imbalanced sample size.*
